# Tumor promoter-induced cellular senescence: cell cycle arrest followed by geroconversion

**DOI:** 10.18632/oncotarget.3011

**Published:** 2014-12-29

**Authors:** Olga V. Leontieva, Mikhail V. Blagosklonny

**Affiliations:** ^1^ Department of Cell Stress Biology, Roswell Park Cancer Institute, Buffalo, NY, USA

**Keywords:** phorbol ester, PMA, TPA, rapalogs, cancer, mTOR, aging, senescence

## Abstract

Phorbol ester (PMA or TPA), a tumor promoter, can cause either proliferation or cell cycle arrest, depending on cellular context. For example, in SKBr3 breast cancer cells, PMA hyper-activates the MEK/MAPK pathway, thus inducing p21 and cell cycle arrest. Here we showed that PMA-induced arrest was followed by conversion to cellular senescence (geroconversion). Geroconversion was associated with active mTOR and S6 kinase (S6K). Rapamycin suppressed geroconversion, maintaining quiescence instead. In this model, PMA induced arrest (step one of a senescence program), whereas constitutively active mTOR drove geroconversion (step two). Without affecting Akt phosphorylation, PMA increased phosphorylation of S6K (T389) and S6 (S240/244), and that was completely prevented by rapamycin. Yet, T421/S424 and S235/236 (p-S6K and p-S6, respectively) phosphorylation became rapamycin-insensitive in the presence of PMA. Either MEK or mTOR was sufficient to phosphorylate these PMA-induced rapamycin-resistant sites because co-treatment with U0126 and rapamycin was required to abrogate them. We next tested whether activation of rapamycin-insensitive pathways would shift quiescence towards senescence. In HT-p21 cells, cell cycle arrest was caused by IPTG-inducible p21 and was spontaneously followed by mTOR-dependent geroconversion. Rapamycin suppressed geroconversion, whereas PMA partially counteracted the effect of rapamycin, revealing the involvement of rapamycin-insensitive gerogenic pathways. In normal RPE cells arrested by serum withdrawal, the mTOR/pS6 pathway was inhibited and cells remained quiescent. PMA transiently activated mTOR, enabling partial geroconversion. We conclude that PMA can initiate a senescent program by either inducing arrest or fostering geroconversion or both. Rapamycin can decrease gero-conversion by PMA, without preventing PMA-induced arrest. The tumor promoter PMA is a gero-promoter, which may be useful to study aging in mammals.

## INTRODUCTION

The mTOR (Target of Rapamycin) signaling pathway is activated by nutrients (glucose, amino and fatty acids), growth factors, cytokines, oxygen, hormones and many other signals [1-4]. In turn, mTOR stimulates cellular size growth and metabolism as well as differentiation-specific functions [3-19]. In cycling cells, mTOR drives mass growth. If the cell cycle is arrested, then mTOR drives “futile growth” or geroconversion, converting reversible arrest to irreversible senescence [5, 20-22]. Senescence is not just cell cycle arrest: arrested cells can be either quiescent or senescent [21-25]. In quiescent cells, mTOR is deactivated [20, 26-33]. For example, serum withdrawal deactivates mTOR and MEK/MAPK pathways, causing reversible quiescence in normal cells [20, 26, 34-36]. In contrast, in senescent cells, mTOR is active [26, 29, 30, 33, 37- 40] Senescent cells are characterized by a large flat morphology (hypertrophy), active metabolism, differentiation-specific hyper-functions, and irreversible loss of proliferative potential [21, 23, 39, 41-58]. A senescent program includes 2 steps: (a) cell cycle arrest and (b) conversion from arrest to senescence [22]. For example, p21 can arrest cell cycle but does not inhibit mTOR. Therefore, mTOR drives geroconversion from p21-induced arrest to senescence. Since mTOR is fully active in cell culture (high levels of mitogens, nutrients and oxygen), it is usually sufficient for a cell to get arrested, in order to become senescent [22]. Rapamycin (and other rapalogs), certain tumor suppressors, including p53, serum-withdrawal, hypoxia and contact inhibition all suppress geroconversion by deactivating mTOR [19, 28, 59-71], thus maintaining quiescence instead. And vice verse, growth factor receptors, Ras, Raf, MEK, PI3K and Akt, which all activate the mTOR/S6K/S6 pathway, are involved in cellular senescence and cancer [72-76]. They are gerogenes, driving gerogenic conversion and oncogenic transformation [21, 64]. We can predict that activators of these pathways will promote both cancer and aging. Phorbol ester is the most well known tumor promoter, which activates MEK/ERK and mTOR/S6K signaling pathways [77-85].

Depending on the cellular context, PMA can cause either cell cycle progression or cell cycle arrest by inducing both cyclin D1 and p21 via the MEK/ERK pathway [43, 86-88]. Cell cycle arrest by itself can lead to senescence, if mTOR is not inhibited. Furthermore, the ability to activate mTOR predicts that PMA may be gero-promoter (promote geroconversion). Accordingly, it can cause cellular senescence, first by arresting cell cycle and then by converting this arrest to senescence (geroconversion). Cell cycle arrest caused by PMA is well studied. For example in SKBR3 cells, PMA over-activates MEK/ERK/MAPK, which in turn induces p21 and cell cycle arrest [86]. Here we show that cells become senescent, because mTOR is constantly active in SKBR3 cells. By blocking geroconversion, rapamycin rendered PMA-treated cells quiescent but not senescent. We also investigated cell lines that are completely resistant to PMA-induced arrest. In these cell lines, arrest was caused by either ectopic p21 or by serum starvation. In these cases, PMA increased geroconversion. Use of three cellular models demonstrated that, regardless of its ability to provoke senescence by arresting cell cycle (first step), PMA also empowers a second step of a senescent program: geroconversion.

## RESULTS

### PMA-induced senescence in SKBR3 cells

**Figure 1:**
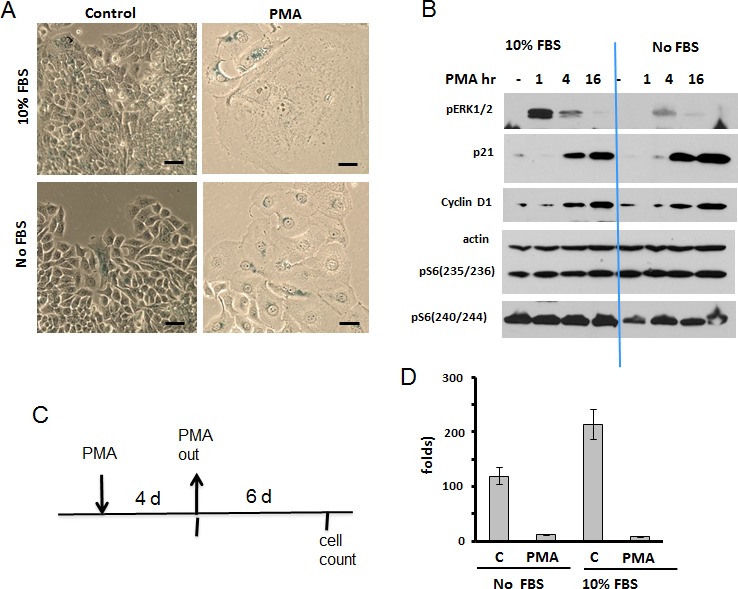
PMA-induced senescence in SKBr3 cells A. Beta-gal staining. SKBR3 cells were treated with 100 nM PMA either in serum-free or in complete (10% FBS) medium. After 4 days drug was washed out and cells were cultured in drug-free medium and stained for beta-gal. Bar – 100 μm. B. Immunoblot analysis. SKBR3 cells were treated with 100 nM PMA in either serum-free or complete medium for times indicated and lysed. Results shown were obtained from 2 separate gels. C-D. RP (reversibility potential) of SKBR3 treated with PMA. C – Schema of experiment; D – RP: SKBR3 cells were plated at low density and treated with 100 nM PMA either in serum-free medium or in complete medium (10% FBS). After 4 days drug was washed out and cells were incubated in drug-free complete medium (10% FBS) for 6 days and counted. Fold increase in cell number was calculated relative to initially plated numbers. Data presented as mean ±SD from triplicates.

As it was investigated in detail in SKBR3 cells [86], PMA activates the MEK/ERK pathway, which in turn induces both p21 and cyclin D1, causing G1 and G2 cell cycle arrest. As it was shown later, hyper-accumulation of cyclin D1 in arrested cells is a marker of senescence [39, 88]. Therefore we checked whether PMA-arrested cells acquire senescent morphology (Fig. 1A). We found that PMA caused a large flat morphology with nucleoli enlargement and beta-Gal positivity (Fig. 1 A). Next, we treated cells with PMA in the absence of serum, expecting that serum withdrawal might inhibit the mTOR pathway and prevent senescence. However, PMA caused senescence both in the presence and absence of the serum. In agreement with previous report [86], PMA rapidly activated ERK1/2 followed by p21 and cyclin D1 induction (Fig. 1 B). We also measured phosphorylation of S6 at S235/236 and S240/244 sites, as markers of mTOR activity. Noteworthy, S235/236 sites are phosphorylated by S6K (a substrate of mTOR) and by RSK (MEK-dependent), whereas S240/244 sites are presumably phosphorylated by S6K only [77-85]. Levels of p-S6 were high in both proliferating cells and serum-starved cells and become even higher after PMA treatment (Fig. 1B). First, this explains why arrested SKBR3 cells become senescent and, second, why they become senescent both in the presence and absence of serum.

These senescent cells lost the reversibility or regenerative potential (RP). In fact, PMA-treated SKBR3 cells poorly proliferated after PMA was washed out (Fig. 1 C, D) (Note: PMA is known to be poorly washable. Yet, even without washing, PMA-induced p21 disappears by day 3 [86] and this is a functional equivalent of washing PMA out).

### Rapamycin suppresses geroconversion in PMA-arrested cells

**Figure 2:**
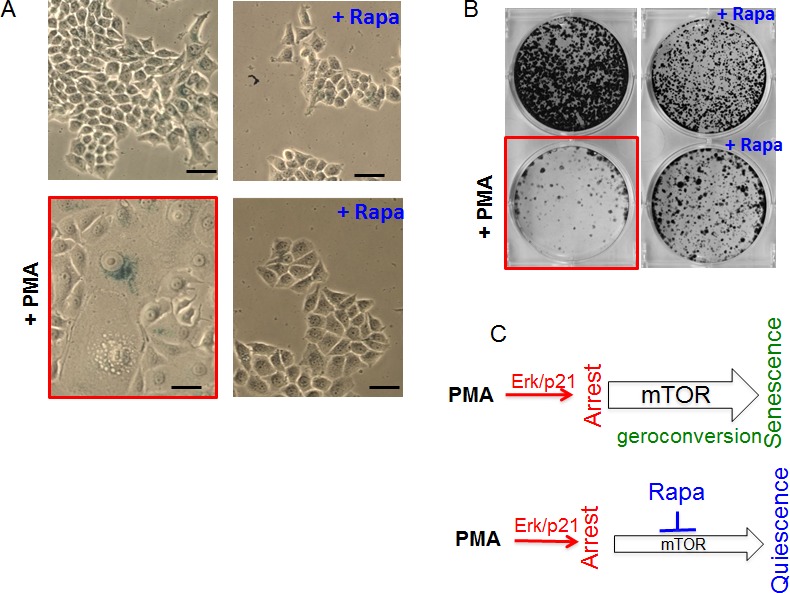
Suppression of PMA-induced senescence by rapamycin in SkBR3 cells A. Beta-gal staining. SkBR3 cells were treated with PMA +/− rapamycin (20 nM) for 5 days, then drugs were washed out and cells were cultured for another 3 days and stained for beta-gal. Bar – 100 μm. B. RP (reversibility potential). SkBR3 cells were plated at low density and treated with 100 nM PMA −/+ rapamycin (20 nM). After 4 days cells were washed and colonies were allowed to regrow in drug-free medium and stained with Crystal Violet after 13 days in culture. C. Schema: PMA-induced senescence and its suppression by rapamycin (Rapa).

As shown in Fig. 2A, PMA caused typical senescent morphology in 30% of SKBR3 cells. Rapamycin by itself slightly inhibited proliferation but did not cause senescent morphology (Fig 2A and Fig. S1). Importantly, rapamycin abrogated PMA-induced senescent morphology (Fig. 2A). We also determined the reversibility potential by the ability of PMA-treated cells to form colonies in drug-free medium. The ability to restart proliferation or RP was decreased in PMA-pretreated cells, measured when PMA was washed out (Fig. 2 B). Rapamycin increased the number of colonies approximately 5-fold (Fig. 2B). We also excluded that rapamycin forced PMA-treated cells to proliferate in the presence of PMA. (Fig. S1A). As expected, co-addition of rapamycin and PMA inhibited proliferation (Fig. S1A). Also, rapamycin alone inhibited proliferation (Fig. S1), whereas inhibiting proliferation, rapamycin prevented PMA-induced loss of the potential to proliferate or RP. As emphasized previously, “proliferation” and “potential to proliferate” should not be confused. Rapamyin never induces proliferation but preserves the potential. Perhaps terms “regenerative potential (RP)”, “reversibility potential (RP)”, “the potential”, “reversibility” should be used to distinguish “proliferative potential” from “proliferation”. So rapamyin suppressed senescent morphology, hypertrophy and maintained reversibility potential (RP). In other words, rapamycin suppressed conversion from reversible arrest to senescence (geroconversion) (Fig. 2C).

### Rapamycin partially abrogates PMA-induced hyper-activation of mTOR targets

**Figure 3:**
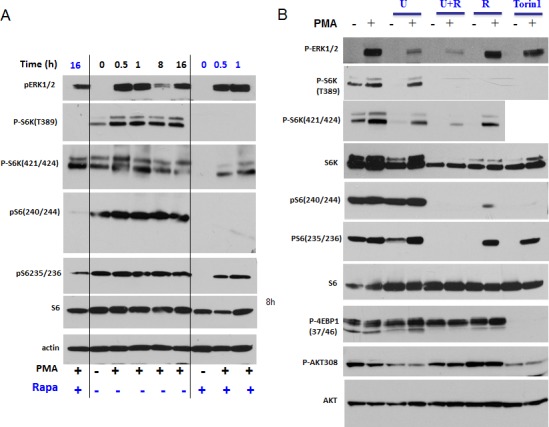
PMA-induced activation of the mTOR pathway in SkBr3 cells A. Immunoblot analysis. SkBR3 cells were treated with 100 nM PMA for the times indicated and lysed. One set was pre-treated (and co-treated) with 100 nM rapamycin for 16 h before adding PMA, as indicated at the bottom (+ Rapa). B. Immunoblot analysis. SkBR3 cells were pre-treated and co-treated with either 10 μMU0126 (U), rapamycin 100 nM (R) or their combination, or with Torin 1 (100 nM) for 24 h. Then 100 nM PMA was added for 1 h and cells were lysed.

PMA induced p-ERK1/2 and p-S6K in both isoforms p70 and p85 (T412) (Fig. 3A). As expected, rapamycin did not affect PMA-induced phosphorylation of ERK1/2. Rapamycin abrogated p-S6K at T389 and p-S6 at S240/244 both in the absence and presence of PMA (Fig. 3A). Also, rapamycin completely eliminated phospho-T421/S424-S6K and phospho-S235/236 -S6 in the absence of PMA. However, rapamycin only marginally affected levels of phospho-T421/S424-S6K and phospho-S235/236 -S6 in the presence of PMA. In other words, PMA caused phosphorylation of S6K and S6 at these sites, even in the presence of rapamycin. This indicates that PMA activates S6K and S6 phosphorylation in part independent of mTOR.

### Both mTOR and MEK pathways are sufficient to phosphorylate S6

**Figure 4:**
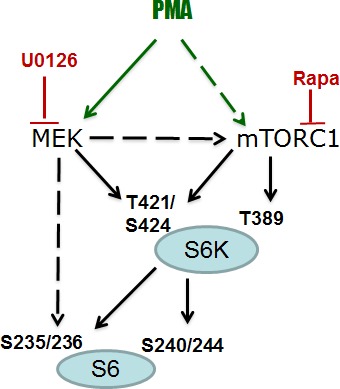
PMA-activated pathways

Since PMA activates the MEK/ERK pathway, we investigated whether inhibition of MEK can prevent PMA-induced phosphorylation of S6K and S6. PMA alone stimulated phosphorylation of ERK1/2 (dramatically), S6K (moderately) and only marginally S6, because S6 has been already near-maximally phosphorylated in proliferating untreated SKBR3 cells (Fig. 3B). Also, SKBR3 cells were treated with PMA in the presence of either U0126 (U), rapamycin (R), U+R or Torin 1 (an inhibitor of both TORC1 and TORC2). As expected, U0126, an inhibitor of MEK, abrogated PMA induced p-ERK1/2 but did not affect p-S6K and p-S6 at all sites tested (Fig. 3B). In contrast, rapamycin abrogated p-S6K (at both sites) and p-S6 (at both sites) in the absence of PMA-stimulation. Yet in the presence of PMA, while abrogating phospho-T389-S6K and phospho-S240/244-S6, rapamycin did not abrogate PMA-induced rapamycin-resistant (RR sites, for brevity) sites: namely, phospho-T421/S424-S6K and phospho-S235/236-S6 sites. Interestingly, Torin 1, which inhibited both complexes of mTOR and prevented phosphorylation of all substrates of mTORC1, as shown for phospho-4EBP1 (T37/46), failed to affect RR sites in the presence of PMA (Fig. 3B). In other words, PMA caused phosphorylation of RR sites in the presence of either rapamycin, U0126 or Torin 1 (Fig. 3B). Only a combination of U+R prevented PMA-induced RR sites (phospho-T421/S424-S6K and phospho-S235/236-S6). We conclude that, in the presence of PMA, S6K can be fully phosphorylated at T421/S424 via mTORC1 (rapamycin sensitive) and via MEK (rapamycin-insensitive) pathways (Fig. 4). Similarly, S6 can be fully phosphorylated on S235/236 via mTORC1 (rapamycin sensitive) and via MEK (rapamycin-insensitive) pathways in the presence of PMA (Fig. 4). Noteworthy, PMA did not cause AKT (T308) phosphorylation. Rapamycin increased Akt phosphorylation (Fig. 3B). This suggests that Akt itself (unlike mTORC1) does not empower geroconversion.

**Figure 5:**
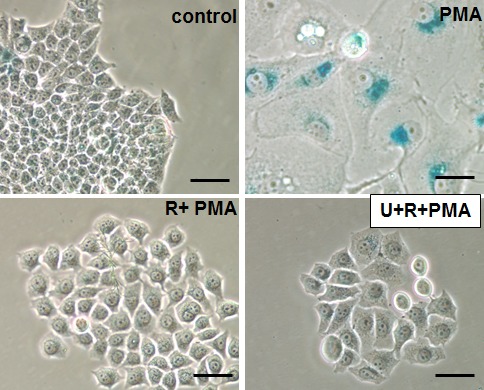
Effects of rapamycin plus U0126 on senescent morphology Beta-gal staining. SkBR3 cells were pre-treated with rapamycin (100 nM) or its combination with U126 (10 μM) for 24 h before adding 100 nM PMA. After 3-day treatment with PMA drugs were washed out and cells were cultured for another 3 days in drug-free medium and stained for beta-gal. Bar – 100 μm.

We emphasize that rapamycin only partly suppressed geroconversion. We investigated whether PMA-induced phospho-T421/S424-S6K and phospho-S235/236 S6 contribute to geroconversion in the presence of rapamycin. As we discussed, addition of U0126 to rapamycin eliminated PMA-induced RR phosphorylation (Fig. 3B). However, this combination did not further suppress senescent morphology in comparison to rapamycin alone (Fig. 5).

### PMA increased geroconversion in HT-p21 cells arrested by p21

**Figure 6:**
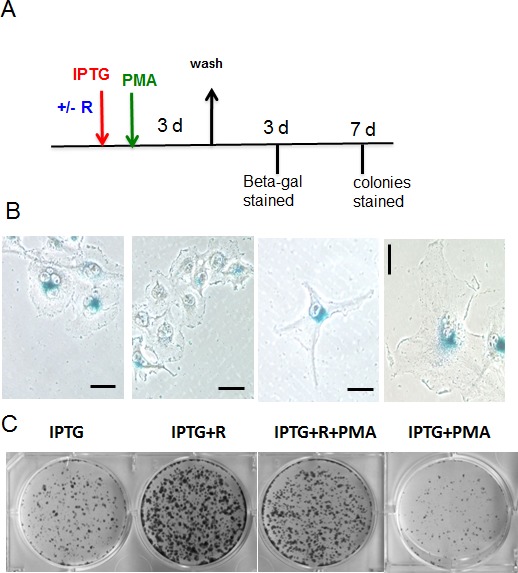
Effects of PMA on IPTG-induced senescence in HT-p21 cells A. Schema of experiment. Rapamycin – R. B-C. HT-p21 cells were plated at low density and treated with IPTG, rapamycin (R) (500 nM) and PMA (100 nM) as indicated in Schema (A). After 3 days drugs were washed out, cells were incubated in drug-free medium for another 3 days and stained for beta-gal (B) (bar – 100 μm) and colonies were stained 7 days after wash (C). As indicated in the Schema, rapamycin was added 1 h before PMA.

We next investigated whether PMA contributes to senescence independently of cell cycle arrest. In HT-p21 model, cell cycle arrest was induced not by PMA but by IPTG-inducible p21 (Fig. 6A). In agreement with previous reports [89], a transient (for 4 days) induction of p21 led to cellular senescence, as evident by senescent morphology (Fig. 6 B). Rapamycin partially suppressed geroconversion to senescent morphology; rapamycin decreased cell size and beta-Gal-staining (Fig. 6 B). In addition, rapamycin preserved the reversibility potential (RP) measured by the ability to form colonies after removal of IPTG. (Note once again: The potential to proliferate (the reversibility potential) should not be confused with proliferation. Thus, rapamycin did not abrogate IPTG-induced arrest but instead preserved the potential to proliferate, when IPTG was washed out).

As shown in Figure S1B, PMA transiently phosphorylated RR sites in the presence of rapamycin. In agreement with the appearance of phospho-S6, this treatment affected geroconversion, increasing the number of senescent cells (morphology) and decreased RP (Fig. 6 B, C).

**Figure 7:**
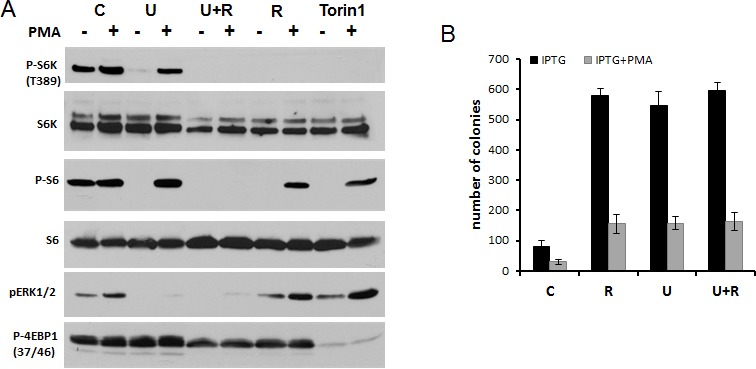
PMA induced rapamycin-insensitive p-S6 in HT-p21 cells A Immunoblot analysis. HT-p21 cells were pre-treated with IPTG in the presence of either rapamycin (500 nM), U126 (10 μM) or their combination or torin 1 (100 nM) for 24 h, then 100 nM PMA was added for 1 h and cells were lysed. All treatments were performed in the presence of IPTG to match conditions shown in fig. 6 and panel 7B. B. RP: HT-p21 cells were pre-treated with IPTG in the presence of different drugs as in panel A for 24 h, then 100 nM PMA was added. After 3 day-treatment with PMA (4 days with IPTG and other drugs), drugs were washed out and colonies were allowed to grow and stained with Crystal violet after 9 days in culture and counted in triplicates. Data are presented as mean ± SD. C – cells treated with IPTG alone; R – treated with IPTG in the presence of rapamycin; U – treated with IPTG in the presence of U126; U+R – treated with IPTG in the presence of combination of rapamycin and U126.

We next investigated induction of p-S6(S235/236) by PMA in detail. Basal levels of phospho-S6K (T389), phospho-S6(S235/236) and phospho-ERK1/2 were high and, therefore, the effect of PMA was marginal (Fig. 7 A). Inhibitor of MEK U0126, inhibitor of TOR rapamycin and inhibitor of TOR kinase Torin 1 all eliminated basal level of phospho-S6K(T389)/phospho-S6(S235/236). This is in agreement with data obtained in SKBR3 cells. Impressively, none of these inhibitors prevented the induction of phospho-S6 (S235/236) by PMA. Only a combination of U0126 and Rapamycin prevented induction of phospho-S6(S235/236) by PMA. These data support the model shown in figure 4.

In HT-p21 cells, both rapamycin and U0126 suppressed geroconversion, as evidenced by preservation of RP (Fig. 7B). Yet, rapamycin and U0126 did not have any additive effect (Fig. 7B). This indicates that PMA-induced phosphorylation of S6 at S235/236 sites is not sufficient by itself to promote geroconversion. Importantly, PMA increased geroconversion both in the absence or presence of rapamycin (Fig. 6 C), suggesting that mTORC1-dependent and -independent pathways are involved in geroconversion.

### PMA-induced geroconversion in quiescent RPE cells

**Figure 8:**
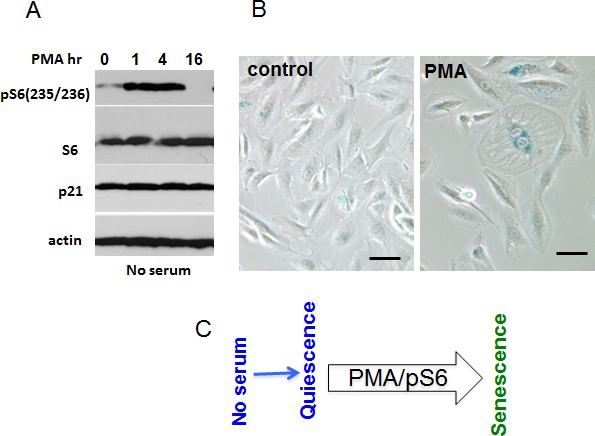
mTOR-dependent geroconversion in RPE cells by PMA A. Immunoblot analysis. RPE cells were incubated in serum-free MEM overnight and then treated with 100 nM PMA for the times indicated. B. Beta-gal staining. RPE cells were pre-incubated in serum free medium before being treated with 100 nM PMA. After 2 day-treatment PMA was washed out, cells were incubated in drug-free medium for another 2 days and stained for beta-gal. Bar – 100 μm. C. Mechanism.

Next, we investigated PMA-induced geroconversion in normal human retinal pigment epithelial (RPE) cells, arrested by serum starvation (Fig. 8). In RPE cells, serum withdrawal causes reversible quiescence, characterized by low levels of p-S6 [26, 34]. In quiescent cells arrested by serum starvation, PMA transiently induced phospho-S6 (Fig. 8 A). PMA did not induce proliferation but instead induced “futile growth” or geroconversion”. This geroconversion is manifested by hypertrophy and beta-Gal staining in approximately 20% of cells, observed after re-addition of serum (Fig. 8 B).

## DISCUSSION

Growth-promoting pathways such as the PI-3K/mTOR pathway are involved in both cancer and aging [21, 64, 90, 91]. Rapamycin prevents age-related diseases and cancer in mammals, including humans [92-116]. Therefore, inhibitors of mTOR are both tumor suppressors and gero-suppressors. To study aging in accelerated fashion, it would be useful to identify “antipode for rapamycin”, an agent that promotes geroconversion. Such agent is expected to (a) activate mTOR and related pathway, (b) be a tumor-promoter. It is known that PMA, a classic tumor-promoter, activates mTOR and MAPK pathways. Importantly, rapamycin can suppress tumor-promotion caused by PMA [117-119].

Given that PMA activates the mTOR pathway, we predicted that PMA can accelerate geroconversion in cell culture. Here we showed that phorbol ester indeed displayed the gero-converting activity. This activity can be obscured by the ability of PMA to initiate senescence, simply by inducing cell cycle arrest. In SKBR3 cells, strong activation of MEK/ERK pathway by PMA causes induction of p21 and cell cycle arrest. When the cell cycle was arrested, still active mTOR pathway drove geroconversion from arrest to senescence. So in SKBR3 cells, PMA caused cell cycle arrest, which was sufficient to cause senescence in the presence of active mTOR. This is consistent with the model of two-step senescence program: cell cycle arrest by PMA plus geroconversion by active mTOR. The mTOR pathway was constitutively activated in cancer SKBR3 cells even in the absence of serum. Rapamycin decreased geroconversion, indicating that mTOR indeed is involved in PMA-induced senescence.

To elucidate the role of PMA in geroconversion, we used the model of IPTG-induced senescence, HT-p21 cells. In this model IPTG, not PMA, caused p21 induction and cell cycle arrest. PMA increased senescence in this cell model, acting as an enhancer of geroconversion. Rapamycin partially decreased geroconversion in the presence and absence of PMA. Yet, PMA still enhanced geroconversion in the presence and absence of rapamycin. This indicates that geroconversion involves some rapamycin-insensitive pathways (in addition to rapamycin-sensitive pathways), which are activated by PMA and are involved in geroconversion. Noteworthy, rapamycin-insensitive phosphorylation of S6K(T421/S424) and S6(S235/236) was also mTOR -independent because Torin 1 (a direct inhibitor of both mTORC1 and mTORC2) did not abrogate-rapamycin insensitive phosphorylation of S6(S235/236) and S6K(T421/S424). We identified pathways that led to rapamycin-insensitive S6K(T421/S424) phosphorylation by PMA (Fig. 4). In agreement with previous reports, PMA induced phosphorylation of S6K and S6 at both rapamycin-sensitive and -insensitive sites in all 3 cell lines tested here. In part, PMA-induced rapamycin-insensitive phosphorylation was dependent on the MEK pathway. Either MEK or mTOR was sufficient to phosphorylate these sites of S6K and S6. Thus, neither rapamycin nor U0126 inhibited phosphorylation of S6K and S6 at T421/S424 and S235/236, respectively, whereas a combination of U0126 and rapamycin eliminated phosphorylation of S6K and S6 on these sites. Yet (and importantly), the addition of U0126 to rapamycin had no effect on geroconversion. This indicates that rapamycin-insensitive phosphorylation of these sites alone is not sufficient to cause geroconversion, when mTORC1 is inhibited. In turn, this indicates that, although rapamycin sensitive-pathways are involved in geroconversion, some unidentified rapamycin-insensitive pathways also contribute to geroconversion. So identification of such pathways remains a challenge.

In conclusion, PMA possesses two senescence-promoting activities: cell cycle arrest (in some cell lines such as SKBR3) and geroconversion. When the cell cycle is arrested by other condition (IPTG-induced p21 or serum starvation), then the geroconverting activity of PMA becomes apparent.

This study further validates the utility of two-step model of senescence for identification of agents which can promote or in contrast suppress aging.

## MATERIALS AND METHODS

### Cell lines and reagents

SKBR3, breast adenocarcinoma cell line (ATCC), was cultured in high-glucose DMEM (-pyruvate) with 10% FBS. HT-p21 cells, derived from HT1080 human fibrosarcoma cells (ATCC, Manassas, VA) were previously described [20, 120, 121] and were cultured in high-glucose DMEM without pyruvate plus 10% FC2 serum (HyClone FetalClone II from Thermo Scientific, Logan, Utah). In these cells, p21 can be turned on or off by isopropyl-thio-galactosidase (IPTG) [20, 120, 121]. Normal retinal pigment epithelial RPE cell line (ATCC, Manassas, VA) was maintained in MEM plus 10% FBS. IPTG was purchased from Invitrogen (Grand Island, NY). Rapamycin was obtained from LC Laboratories (MA, USA). U0126 and PMA were from Sigma-Aldrich (St. Louis, MO). Torin 1 was obtained from Selleck chemicals LCC (Houston, TX).

### SA-β-Gal staining

Beta-Gal staining was performed using Senescence-galactosidase staining kit (Cell Signaling Technology) according to manufacturer's protocol. Cells were incubated at 37^o^C until beta-gal staining becomes visible. Development of color was detected under light microscope.

### Immunoblot analysis

Whole cell lysates were prepared using boiling lysis buffer (1% SDS, 10 mM Tris.HCl, pH 74.). Equal amounts of proteins were separated using Criterion or mini gradient polyacrylamide gels (Bio-Rad, Hercules, CA) and transferred to PVDF membranes. The following rabbit antibodies for: phospho-S6 (Ser235/236 and S240/244), phospho-AKT (T308), phospho ERK ½, AKT, phospho-4EBP1(T37/46) and phospho-S6K(T421/S424)- were from Cell Signaling Biotechnology (Danvers, MA). Mouse anti-phospho-Thr 389 -S6K and anti-S6 antibody were from Cell Signaling Biotechnology. Rabbit anti-actin antibody was from Sigma-Aldrich (St. Louis, MO); mouse antibodies for p21 and cyclin D1 were from from BD Biosciences (San Jose, CA) and Santa Cruz Biotechnology (Paso Robles, CA), respectively. Secondary anti-rabbit and anti-mouse HRP-conjugated antibodies were from Cell Signaling Biotechnology.

### RP (reversibility potential)

Cells were plated at low densities, treated with senescence inducing drugs for 3-4 days as indicated in figure legends. Then, drugs were washed out and cells were allowed to re-grow in fresh drug-free medium for a few days (as indicated in figure legends). Then cells were either counted or formed colonies were stained with 1% Crystal Violet (Sigma-Aldrich) and counted.

## SUPPLEMENTARY MATERIAL AND FIGURES


