# Supplementary material for: Tumor promoter-induced cellular senescence: cell cycle arrest followed by geroconversion
Source: Oncotarget. 2014 Dec 29;5(24):12715–27. doi: 10.18632/oncotarget.3011 (PMC4350340; doi:10.18632/oncotarget.3011)
Supplement: Supplementary file 1 [file oncotarget-05-12715-s001.pdf]

Tumor promoter-induced cellular senescence: cell cycle arrest followed by geroconversion

Supplementary Material

A

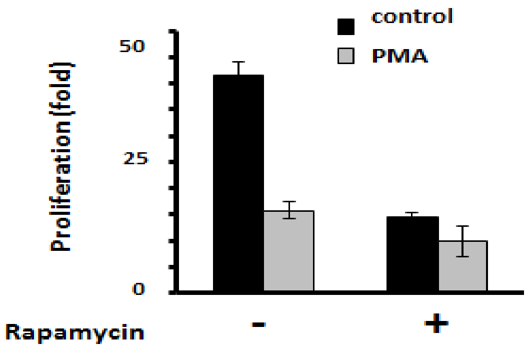

Supplement Fig S1A: Effects of PMA and rapamycin on proliferation of SKBR3 cells. Cells were treated with 100 nM PMA in the presence or absence of 50 nM rapamycin for 4 days and counted. Data presented as fold increase in cell numbers compared to initially plated number ± SD.

B

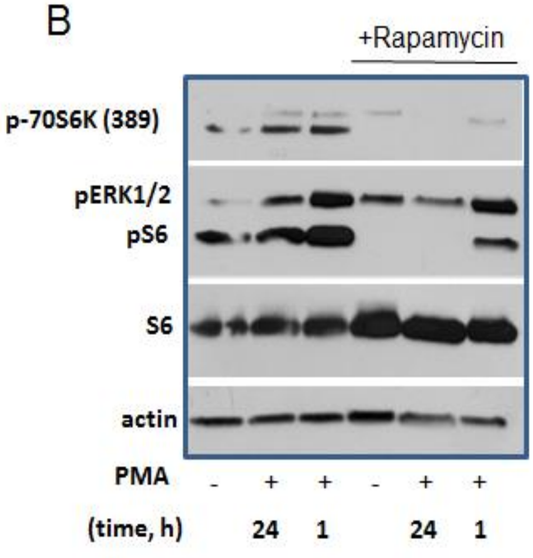

Supplement Fig S1B: Immunoblot analysis. One set of HT-p21 cells was pre-treated with IPTG +/- 500 nM rapamycin for 2 h before 100 nM PMA was added and cells were lysed after 24h-treatment with PMA. 2nd set was pre-treated with IPTG +/- rapamycin for 24 h and then PMA was added and cells were lysed after 1h-treatment with PMA.
